# ABCA7 high‐risk genotype predicts working memory decline among older cognitively healthy African Americans

**DOI:** 10.1002/alz.091452

**Published:** 2025-01-09

**Authors:** Mustafa Sheikh, Wiktoria Piaszczynska, Alyssa Arnasalam, Arcande Oyono, José Mojica Perez, Miray Budak, Bernadette A. Fausto, Mark A. Gluck

**Affiliations:** ^1^ Rutgers University–Newark, Newark, NJ USA

## Abstract

**Background:**

Alzheimer's disease (AD) is a neurodegenerative disease characterized by progressive cognitive decline^1^. APOE‐ε4 has been identified as the most prevalent genetic risk factor for the early onset of AD, while ABCA7‐80 (rs115550680) has been shown to have a stronger effect size than the APOE‐ε4 allele and is associated with the development of late‐onset of AD among African Americans^2,3^. Although the efficiency of executive functions declines with age, some basic attentional functions and preserved knowledge may help mitigate the effects of aging on working memory^4^. Nevertheless, the impact of APOE ε4 and ABCA7‐80 genotypes on attention and executive function in preclinical AD remains unclear^5^. This study investigated the influence of APOE ε4 and ABCA7‐80 genotypes on working memory among cognitively unimpaired older African Americans.

**Method:**

Participants were drawn from the ongoing longitudinal study, Pathways to Healthy Aging in African Americans conducted at Rutgers University–Newark. 838 participants (ages ≥ 60) completed saliva collection for genotyping and neuropsychological battery for cognitive assessment with a fraction repeating each assessment multiple times as part of returning visits. Simple linear mixed models were applied for longitudinal statistical analysis.

**Result:**

ABCA7‐80 high‐risk genotype was found to be a risk factor for decreased cognitive performance, as assessed by Trail Making Test Part A (ß = 5.819, p = 0.030) and Part B (ß = 39.964, p < 0.001), Digit Span Total (ß = ‐2.092, p= 0.045), Controlled Oral Word Association (ß = ‐4.708, p = 0.019), as well as a significant association with increased depression.

**Conclusion:**

The ABCA7‐80 high‐risk genotype may indicate early working memory declines in preclinical AD and may serve as a predictive biomarker among cognitively impaired older African Americans.

